# Epidemiological profile of patients with malignant neoplasm admitted to a tertiary care center in India: a retrospective cross-sectional study

**DOI:** 10.3389/fonc.2025.1636807

**Published:** 2025-12-08

**Authors:** Sourav Debnath, Pusparghya Pal, Anurag Kumar Singh, Shivang Mishra, Sumit Rajotiya, Manashi Ghosh, Prashant Nakash, Sachin Kumar, Roshni Singh, Govind Sharma, Mahaveer Singh, Deepak Nathiya, Balvir Singh Tomar

**Affiliations:** 1 Department of Pharmacy Practice, Nims Institute of Pharmacy, Nims University Rajasthan, Jaipur, India; 2 Department of Medical Oncology, National Institute of Medical Sciences and Research, Nims University Rajasthan, Jaipur, India; 3 Department of Management, Nims Institute of Management and Commerce, Nims University Rajasthan, Jaipur, India; 4 Department of Endocrinology, National Institute of Medical Sciences and Research, Nims University Rajasthan, Jaipur, India; 5 Institute of Pediatric Gastroenterology and Hepatology, National Institute of Medical Sciences and Research, Nims University Rajasthan, Jaipur, India

**Keywords:** cancer epidemiology, rural-urban disparities, treatment access, tobacco use, oncology outcomes, Rajasthan

## Abstract

**Background:**

Cancer poses a growing public health challenge in India, with significant urban–rural disparities in diagnosis and treatment. This study aimed to evaluate the epidemiological profile and treatment patterns of cancer patients in Rajasthan, focusing on differences in disease presentation, treatment modalities, and outcomes between urban and rural populations.

**Methods:**

A retrospective observational study was conducted at the National Institute of Medical Sciences and Research, Jaipur, including 1,366 histopathologically confirmed cancer patients admitted between January 2021 and December 2023. Data on demographics, cancer type, stage, treatment, and outcomes were analyzed using SPSS version 28, comparing rural and urban groups.

**Results:**

Of the 1,366 patients, 77.45% were from rural areas. Rural patients had higher rates of advanced-stage (Stage IV) presentation (56.1% vs. 47.7%, p = 0.047) and tobacco use (16.8% vs. 10.4%, p = 0.006). Head and neck cancers were most common in men (20.7%), and breast cancer in women (8.2%). Chemotherapy was the predominant treatment (84%) modality, while urban patients more frequently received multimodal therapy. In-hospital mortality was comparable between groups (4.82% vs. 4.22%, p = 0.661), as were readmission rates.

**Conclusion:**

Rural patients experienced a greater cancer burden due to delayed diagnosis and limited access to comprehensive care. Strengthening rural oncology services, improving early detection, and addressing modifiable risk factors like tobacco use are critical for reducing disparities and improving outcomes.

## Introduction

Cancer remains one of the most formidable challenges to global health, representing a complex disease characterized by uncontrolled cell growth that leads to significant morbidity and mortality. In 2022 alone, approximately 20 million new cancer cases were diagnosed globally, with nearly 9.7 million deaths attributed to this devastating disease. Among the various types, lung cancer (12.4%) emerged as the most frequently diagnosed malignancy, followed by colorectal (9.6%), prostate (7.3%), and stomach cancers (4.9%) (1). Asia, home to nearly half the global population, bore the brunt of the cancer burden, accounting for 49.3% of global cases with an incidence rate of 169.1 per 100,000 in 2020 (2). While historically prevalent in developed nations, the incidence of cancer has seen a dramatic rise in developing countries in recent decades (3).

In India, the scenario is equally alarming. With over 1.32 million new cases and 0.85 million deaths reported in 2022, cancer is now the second leading cause of mortality in the country. The spectrum of cancers affecting the Indian population includes malignancies of the lung, breast, stomach, cervix, and esophagus, among others (4, 5). Striking geographical variations highlight the heterogeneity of cancer prevalence across the country, from the densely populated Ganges belt to the Deccan plains, and from urban centers to rural communities (6). Particularly concerning is Northeast India, where the incidence of cancers such as nasopharyngeal, esophageal, and cervical cancers is disproportionately high (7). Among women, breast cancer is the most prevalent, with an age-adjusted incidence rate of 25.8 per 100,000 and a mortality rate of 12.7 per 100,000 (8). In Rajasthan, the crude annual cancer incidence rose markedly from 58.8 to 72.6 per 100,000 people between 1990 and 2016, with breast cancer prevalence reaching as high as 98 per 100,000 in rural regions (9, 10).

In line with the study objective, this investigation specifically aims to explore the epidemiological distribution and urban–rural disparities among patients with malignant neoplasms in a tertiary care hospital in Rajasthan.

The rising cancer burden in India is driven by a complex interplay of genetic predisposition, environmental factors, and lifestyle choices (11). Beyond its profound health implications, cancer also imposes a significant economic burden, with high costs of diagnosis and treatment further exacerbating the challenges faced by patients and their families (12).

Understanding the epidemiological profile of cancer patients is crucial for formulating effective cancer control strategies. Comprehensive data on the types, distribution, and demographic patterns of malignancies can help guide early detection, screening programs, and prevention efforts tailored to specific regional needs. This study was therefore designed to identify patterns of healthcare utilization, stage at diagnosis, and treatment characteristics among urban and rural cancer patients, thereby addressing a major knowledge gap in regional cancer epidemiology.

## Methodology

### Study design and population

**Figure 1:**
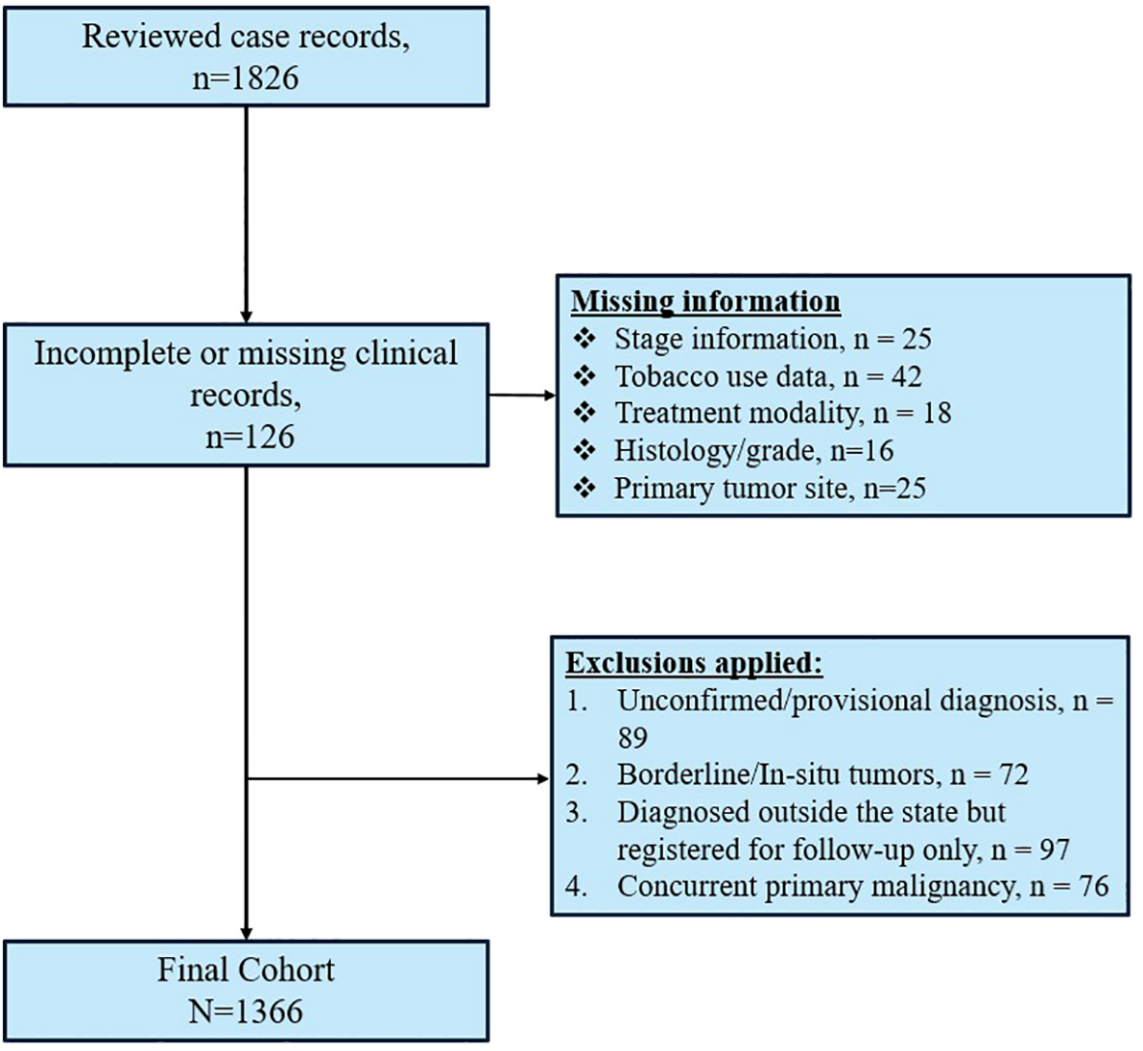
Illustrating case selection and exclusion process for the study.

This study was conducted at the National Institute of Medical Sciences and Research (NIMS), a tertiary care hospital in Jaipur, Rajasthan. A total of 1826 case sheets from the surgical and medical oncology departments were retrospectively reviewed from medical records between January 2021 and December 2023. As this was a retrospective record-based study, all consecutive eligible cases within the defined period were included. No formal sample size calculation was performed. The selection and exclusion process of cases are illustrated in **Figure 1**.

### Inclusion and exclusion criteria

Inclusion criteria were strictly defined to ensure data reliability and clinical relevance. Patients were included if they met all of the following conditions: confirmed histopathological or cytological diagnosis of invasive malignancy (excluding carcinoma *in situ* and borderline tumors); complete medical records including demographic details, clinical staging, diagnostic reports, and treatment documentation; primary cancer diagnosis and initiation of treatment at the participating hospital (to ensure consistency in staging and management protocols); no concurrent primary malignancy at the time of registration; and age ≥ 18 years at diagnosis (all adult age categories included).

Exclusion criteria were applied as follows: patients with unconfirmed cancer diagnosis, incomplete medical records, borderline tumors or intraepithelial neoplasia, those diagnosed outside Rajasthan but registered at NIMS only for follow-up, and patients with coexisting primary cancers were excluded.

After applying these criteria, 1366 patients from 23 districts of Rajasthan (Ajmer, Alwar, Barmer, Bharatpur, Bhilwara, Bikaner, Bundi, Churu, Dausa, Dhaulpur, Hanumangarh, Jaipur, Jalor, Jhunjhunu, Jodhpur, Karauli, Kota, Sawai Madhopur, Nagaur, Pali, Sikar, Shri Ganganagar, Tonk) were included in the final analysis.

### Cancer classification

Cancer types were classified according to the International Classification of Diseases for Oncology, 3rd edition (ICD-10) of the World Health Organization (13). Demographic, clinical, diagnostic, and treatment data were retrieved from hospital records. Tumor staging was performed according to the *American Joint Committee on Cancer (AJCC) 8th edition*, using clinical staging information abstracted from patient medical records by trained research staff based on documentation by the treating oncologists at the time of diagnosis (14).

### Lifestyle and behavioral variables

Information on smoking, alcohol consumption, and smokeless tobacco (chewing) was obtained from patient medical records as documented during hospital admission or outpatient evaluation. Smoking (any form) included the use of any smoked tobacco products such as cigarettes, bidis, or hookah, while tobacco (chewing) referred to smokeless forms such as gutkha, khaini, or betel quid with tobacco. Alcohol use referred to any reported consumption of alcoholic beverages. Only patients with current use of smoking, alcohol, or smokeless tobacco at the time of admission were coded as “Yes,” whereas those without current use were coded as “No”.

### Data analysis

Socio-demographic and clinical characteristics were analyzed using descriptive statistics. Continuous variables were reported as mean ± standard deviation, and categorical variables as frequencies and percentages. Data were stratified by area of residence (rural vs. urban). Normality was assessed using the Shapiro-Wilk test. Group comparisons for continuous variables were performed using the independent Student’s t-test, and for categorical variables using the chi-square test. Statistical significance was set at p < 0.05. Multivariable binary logistic regression was performed to evaluate the independent association between residence status and stage at presentation. All analyses were conducted using Statistical Package for the Social Sciences (SPSS) version 28 software. Key findings were visualized using bar charts and pie charts created in Microsoft Excel for enhanced interpretability.

## Results

### Clinic-demographic characteristics

A total of 1366 patients were enrolled, of whom 1058 (77.45%) were from rural areas and 308 (22.54%) were from urban areas. The mean age was comparable between rural (54.00 ± 15.88 years) and urban (53.05 ± 13.52 years) patients. Age distribution across categories (children, adolescents, adults, and older adults) did not differ significantly between the groups (p = 0.372) (**Table 1**).

**Table 1:** Clinic-demographic characteristics of the patients.

Variables [n (%)]	Rural [1058, (77.45)]	Urban [308, (22.54)]	Total [1366, (100)]	*P*-value
Age, mean ± SD	54.00 ± 15.88	53.05 ± 13.52	53.77 ± 15.25	0.952
Age Category, n (%)				
Child (1–12 years)	18 (1.7)	3 (1.0)	21 (1.53)	0.372
Adolescent (13–17 years)	12 (1.1)	0	12 (0.87)	
Adult (18–59 years)	587 (55.5)	195 (63.3)	782 (57.24)	
Older adults (>59 years)	441 (41.7)	110 (35.7)	551 (40.33)	
Gender, n (%)				
Male	573 (54.15)	148 (48.05)	721 (52.78)	0.063
Female	485 (45.84)	160 (51.94)	645 (47.21)	
Smoking (any form), n (%)				
Yes	320 (30.2)	80 (26.0)	400 (29.28)	0.147
No	738 (69.8)	228 (74.0)	966 (70.71)	
Alcohol, n (%)				
Yes	205 (19.4)	53 (17.2)	258 (18.88)	0.392
No	738 (69.8)	255 (82.8)	993 (72.69)	
Tobacco (chewing), n (%)				
Yes	178 (16.8)	32 (10.4)	210 (15.37)	**0.006**
No	880 (80.6)	276 (89.6)	1156 (84.62)	
Stage, n (%)				
Stage-1	11 (1.0)	1 (0.3)	12 (0.9)	**0.047**
Stage-2	141 (13.3)	50 (16.2)	191 (14)	
Stage-3	312 (29.5)	110 (35.7)	422 (30.9)	
Stage-4	594 (56.1)	147 (47.7)	741 (54.2)	
Length of hospital stay (LHS), median (IQR)	18 (13-23)	18 (12-24)	18 (13-23)	0.657
LHS (Days), n (%)				
01-Dec	269 (25.42)	83 (27.21)	352 (25.76)	0.6
13-24	606 (57.27)	164 (53.24)	770 (56.36)	
25-36	171 (16.16)	54 (17.53)	225 (16.47)	
37-48	11 (1.03)	6 (1.96)	17 (1.24)	
49-60	1 (0.09)	1 (0.32)	2 (0.14)	
In-hospital mortality, n (%)	51 (4.82)	13 (4.22)	64 (4.68)	0.661
Readmission, n (%)				
< 5 times	785 (74.2)	241 (78.2)	1026 (75.1)	0.148
≥ 5 times	273 (25.8)	67 (21.8)	340 (24.9)	

All the values are presented as frequency (%) or mean and standard deviation (SD) otherwise stated. IQR- Interquartile range; *p-value* considered statistically significant at < 0.05. The *p-values* of significant variables were bolded.

“Smoking” refers to use of any smoked tobacco (e.g., cigarettes, bidi, hookah), while “Tobacco use” refers to smokeless forms (e.g., chewing, gutkha, khaini).

Gender distribution showed a slightly higher proportion of females in the urban cohort (51.94%) compared with the rural group (45.84%). The prevalence of smoking (29.28%), alcohol consumption (18.88%), and smokeless tobacco use (15.37%) was assessed, with tobacco chewing significantly higher among rural patients (16.8%) than urban patients (10.4%) (p = 0.006) (**Table 1**).

Disease staging showed a statistically significant difference (p = 0.047), with rural patients more frequently presenting at stage 4 (56.1%) compared with urban patients (47.7%). The median length of hospital stay was identical across both groups (18 days), with interquartile ranges showing minimal variation (rural: 13–23, urban: 12–24, p = 0.657). The majority of patients (56.36%) had hospital stays ranging from 13 to 24 days. Overall clinical characteristics and outcome measures are shown in **Table 1**.

### Multivariable regression analysis

To account for potential confounding, a multivariable binary logistic regression analysis was performed to assess factors associated with late-stage cancer presentation, adjusting for age, sex, residence, tobacco use, and treatment modality. After controlling for these variables, rural residence remained significantly associated with late-stage presentation (AOR = 1.37, 95% CI: 1.06 to 1.78, p = 0.018) (**Supplementary Table S1**).

### Distribution of cancer types

**Figure 2:**
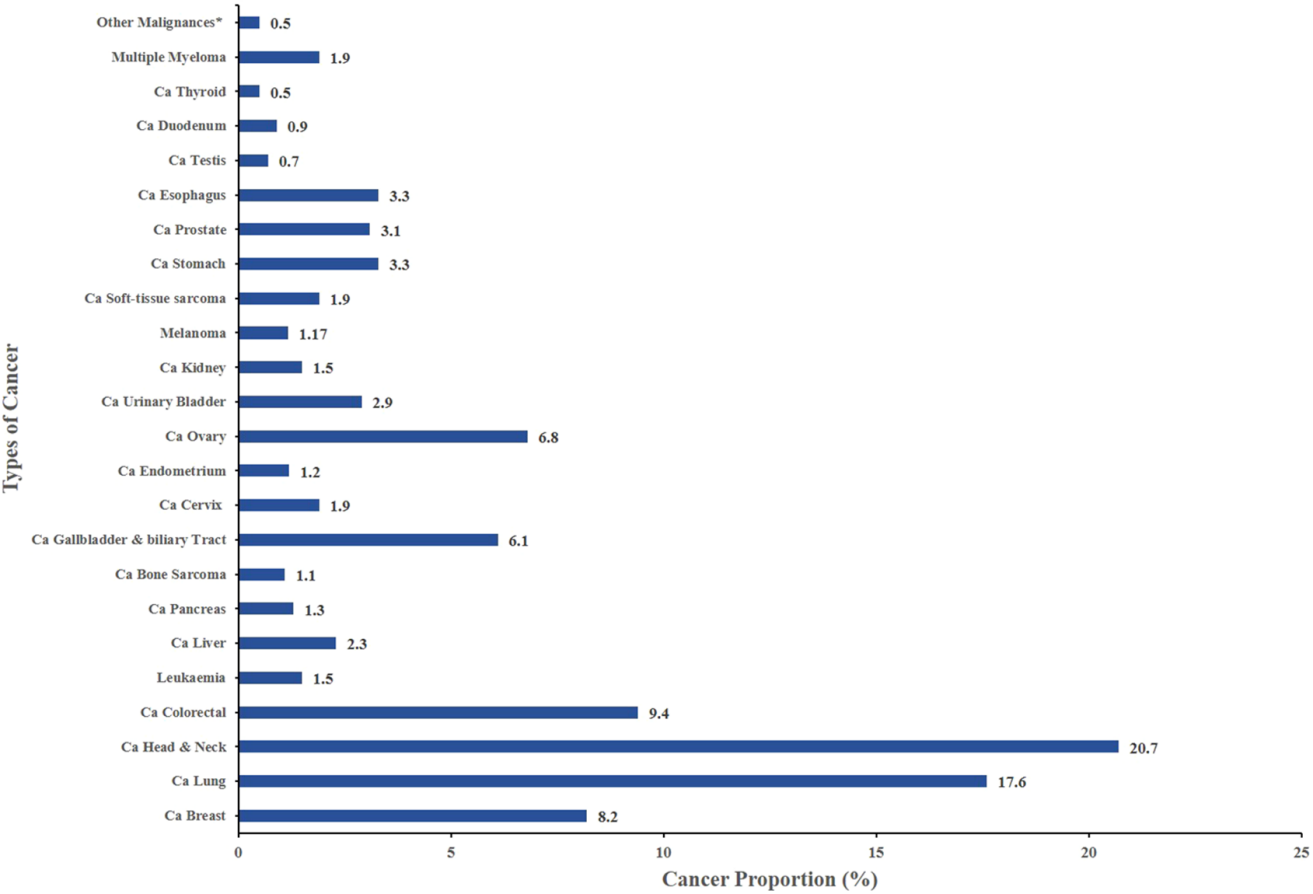
Distribution of different types of malignancies.

In our study cohort, the most commonly diagnosed cancers in men includes Head & Neck Cancer (20.7%), Lung Cancer (17.6%), Prostate Cancer (6.8%), Stomach Cancer (3.3%). In contrast, women predominantly present with breast cancer (8.2%), cervical cancer (6.8%), ovarian cancer (6.1%), Gallbladder & Biliary Tract Cancer (2.9%) reflecting the substantial impact of reproductive and hormonal factors on cancer epidemiology (**Table 2**; **Figure 2**).

**Table 2:** Comparing the incidence of cancer across rural and urban settings by gender.

Variables [n (%)]		Rural [1058, (77.45%)]		Urban [308, (22.5)]		Total [1366, (100)]
Sites of cancer, n (%)	ICD-10	Male [573, (54.2)]	Female [485, (45.8)]	Male [148, (48.1)]	Female [160, (51.9)]	
1. Ca Breast	C50.9	2 (0.3)	74 (15.8)	0	36 (22.9)	112 (8.2)
2. Ca Lung	C34.9	127 (21.5)	64 (13.7)	32 (21.2)	17 (10.8)	240 (17.6)
3. Ca Head & Neck	C76.0	147 (24.9)	73 (15.6)	35 (23.2)	28 (17.8)	283 (20.7)
4. Ca Colorectal	C18.9	61 (10.3)	46 (9.8)	12 (7.9)	10 (6.4)	129 (9.4)
5. Leukemia	C95.0	12 (2.0)	4 (0.9)	1 (0.7)	3 (1.9)	20 (1.5)
6. Ca Liver	C22.0	8 (1.3)	10 (2.1)	7 (4.6)	5 (3.2)	30 (2.1)
7. Ca Pancreas	C25.9	11 (1.9)	4 (0.9)	1 (0.7)	2 (1.3)	18 (1.3)
8. Ca Bone Sarcoma	C41.9	9 (1.5)	4 (0.9)	1 (0.7)	1 (0.6)	15 (1.1)
9. Ca Gallbladder & biliary Tract	C23	33 (5.6)	34 (7.3)	7 (4.6)	10 (6.4)	84 (6.1)
10. Ca Cervix	C53.9	0	23 (4.7)	0	4 (2.5)	27 (1.9)
11. Ca Endometrium	C54.1	0	12 (2.6)	0	4 (2.5)	16 (1.2)
12. Ca Ovary	C56.9	0	71 (14.6)	0	21 (13.4)	92 (6.7)
13. Ca Urinary Bladder	C67.9	16 (2.7)	10 (2.1)	7 (4.6)	5 (3.2)	38 (2.7)
14. Ca Kidney	C64.9	8 (1.4)	5 (1.1)	7 (4.6)	1 (0.6)	21 (1.5)
15. Melanoma	C43.9	8 (1.4)	5 (1.1)	2 (1.3)	1 (0.6)	16 (1.1)
16. Ca Soft-tissue sarcoma	C49.9	9 (1.5)	10 (2.1)	5 (3.3)	2 (1.3)	26 (1.9)
17. Ca Stomach	C16.9	22 (3.7)	17 (3.6)	4 (2.6)	2 (1.3)	45 (3.3)
18. Ca Prostate	C61	34 (5.8)	0	9 (6.0)	0	43 (3.1)
19. Ca Esophagus	C15.9	31 (5.3)	6 (1.3)	7 (4.6)	1 (0.6)	45 (3.3)
20. Ca Testis	C62.9	5 (0.8)	0	5 (3.3)	0	10 (0.7)
21. Ca Duodenum	C17.0	8 (1.4)	3 (0.6)	1 (0.7)	0	12 (0.9)
22. Ca Thyroid	C73	2 (0.3)	2 (0.4)	1 (0.7)	2 (1.3)	7 (0.5)
23. Multiple Myeloma	C90.0	10 (1.7)	8 (1.7)	4 (2.6)	4 (2.5)	26 (1.9)
24. Other Malignances*		10 (1.7)	0	0	1 (0.6)	11 (0.8)

Other Malignances*- Brain Tumor, Ca Penis, Ca Adrenal Gland, Carcinoma of unknown primary.

ICD-10 for Other Malignances- C71.0, C60.9, C74.0, C80.1.

**Table 3** shows the distribution of cancer types across four age categories: children, adolescents, adults, and older adults. The majority of cancer cases occurred in adults (57.39%) and older adults (40.26%), with only a small proportion in children (1.57%) and adolescents (0.8%). The most common malignancies in adults and older adults were breast, lung, head and neck, and colorectal cancers. Pediatric and adolescent cancers were rare, with only isolated cases of leukemia, liver cancer, bone sarcoma, and soft-tissue sarcoma.

**Table 3:** The distribution of cancer cases according to age groups.

Cancer site	Age category (years), n (%)			
	Child (1–12 years) (21)	Adolescent (12–17 year) (11)	Adult (18–59 years) (784)	Older adult (>60 years) (550)
1. Ca Breast	1 (4.8)	0	66 (8.4)	45 (8.2)
2. Ca Lung	1 (4.8)	3 (25.0)	126 (16.1)	110 (20.0)
3. Ca Head & Neck	0	1 (8.3)	168 (21.4)	114 (20.7)
4. Ca Colorectal	1 (4.8)	1 (8.3)	74 (9.5)	53 (9.6)
5. Leukemia	1 (4.8)	0	14 (1.8)	5 (0.9)
6. Ca Liver	2 (9.5)	1 (8.3)	18 (2.3)	10 (1.8)
7. Ca Pancreas	0	0	8 (1.0)	10 (1.8)
8. Ca Bone Sarcoma	0	4 (33.3)	7 (0.9)	4 (0.7)
9. Ca Gallbladder & biliary Tract	1 (4.8)	0	47 (6.0)	36 (6.5)
10. Ca Cervix	0	0	16 (2.0)	11 (2.0)
11. Ca Endometrium	1 (4.8)	0	8 (1.0)	7 (1.3)
12. Ca Ovary	2 (9.5)	0	62 (7.9)	29 (5.3)
13. Ca Urinary Bladder	0	0	22 (2.8)	16 (2.9)
14. Ca Kidney	0	0	9 (1.2)	12 (2.2)
15. Melanoma	0	0	8 (1.0)	8 (1.5)
16. Ca Soft-tissue sarcoma	3 (14.3)	2 (18.2)	13 (1.7)	8 (1.5)
17. Ca Stomach	0	0	24 (3.1)	21 (3.8)
18. Ca Prostate	0	0	23 (2.9)	20 (3.6)
19. Ca Esophagus	2 (9.5)	0	27 (3.5)	16 (2.9)
20. Ca Testis	1 (4.8)	0	7 (0.9)	2 (0.4)
21. Ca Duodenum	1 (4.8)	0	8 (1.0)	3 (0.5)
22. Ca Thyroid	1 (4.8)	0	3 (0.4)	3 (0.5)
23. Ca Myeloma	1 (4.8)	0	20 (2.6)	5 (0.9)
24. Other Malignances*	2 (9.5)	0	4 (0.5)	2 (0.3)

Other Malignances*- Brain Tumor, Ca Penis, Ca Adrenal Gland, Carcinoma of unknown primary.

### District-wise distribution

**Figure 3:**
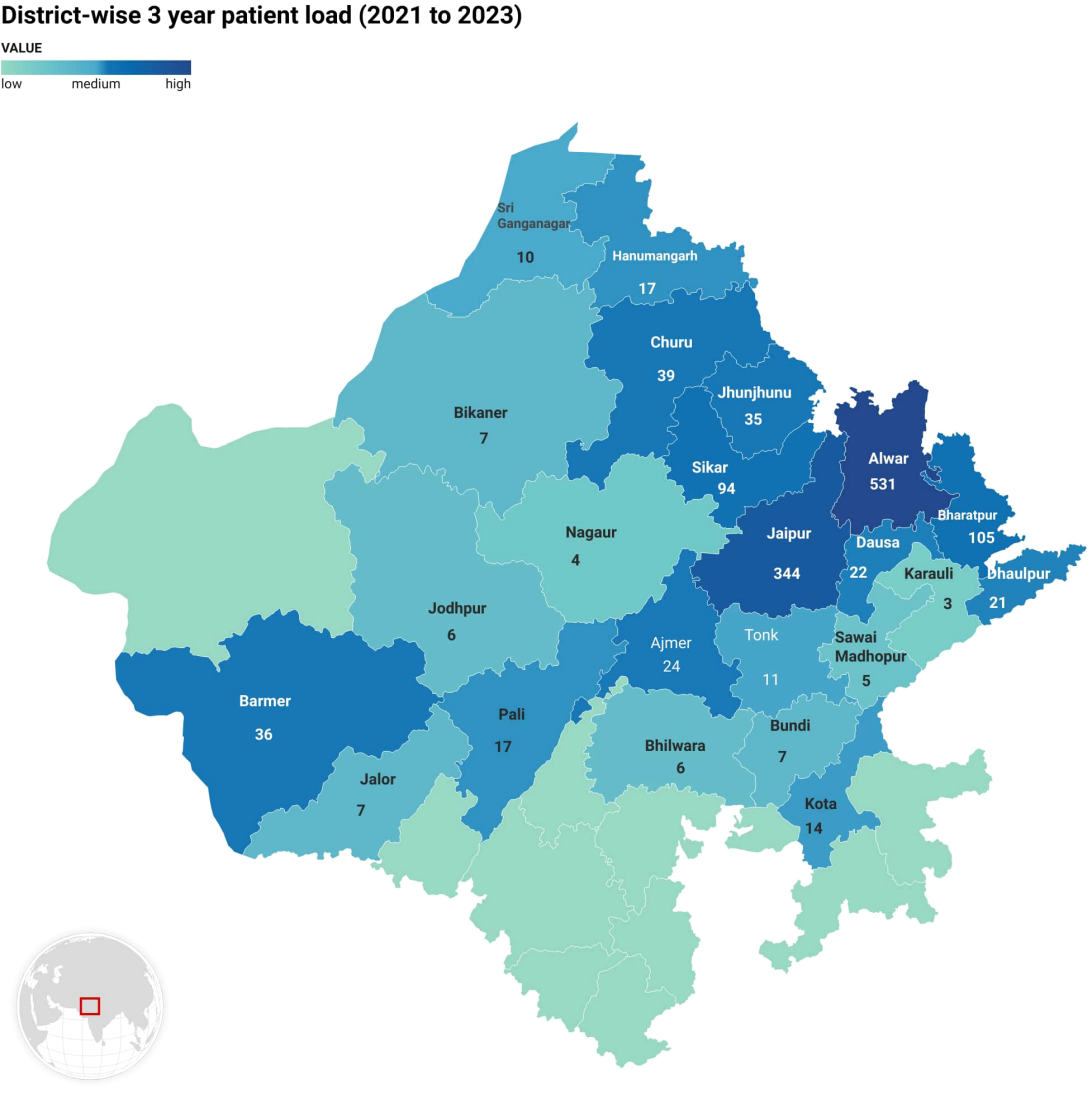
District-wise 3-year patient load.

The highest number of cases were reported in Alwar (38.9%), followed by Jaipur (25.2%), Bharatpur (7.7%), Sikar (7.0%) of the cases, while other districts such as Jalor and Jodhpur, Sawai Madhopur, Karauli contributed smaller proportions (**Figure 3**).

### Treatment modalities

**Figure 4:**
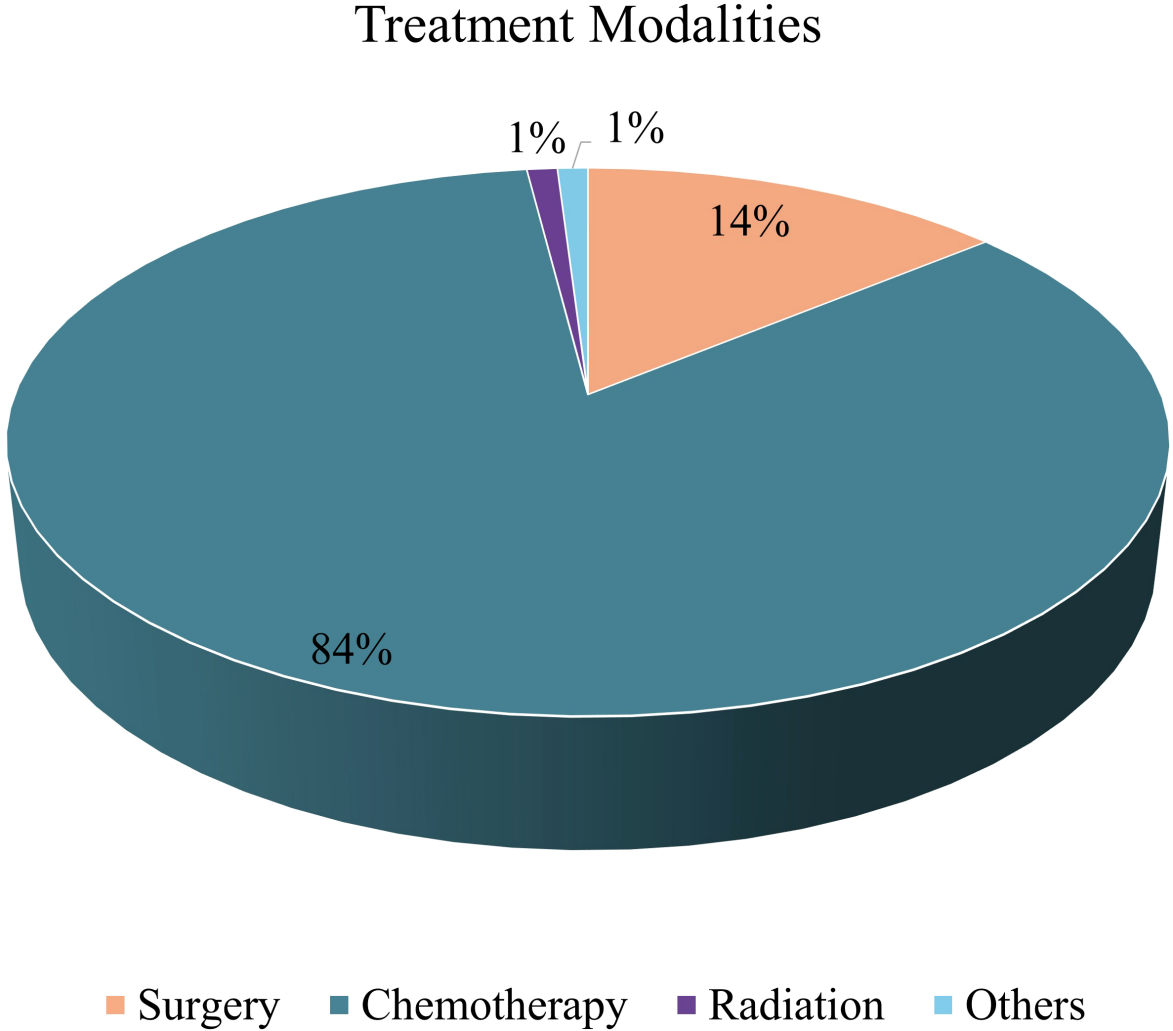
Distribution of treatment modalities among study participants.

**Table 4** outlines how different treatment plans were used for 1,366 patients. Chemotherapy was the predominant treatment modality (84%), followed by surgery (14%), radiation therapy (1%), and other treatments (1%) (**Figure 4**). The lower proportion of radiation therapy cases is attributed to the recent establishment of the Radiation Oncology Department in late October 2023, with data reviewed up to December 2023.

**Table 4:** Distribution of patients across various treatment regimens administered.

Treatments	N (%)
Chemo cycle	
1	83 (6.0)
2	419 (30.6)
3	392 (28.6)
4	134 (9.8)
5	80 (5.8)
6	81 (5.9)
Single regimen	
Paclitaxel	73 (5.3)
Carboplatin	26 (1.9)
Cisplatin	23 (1.6)
Docetaxel	4 (0.2)
Abiraterone	4 (0.2)
Enzaluterone	3 (0.2)
Other Chemo	8 (0.5)
Chemotherapy + Radiation	109 (7.9)
Surgery + Chemotherapy	203 (14.8)
Double regimen	
Paclitaxel + Carboplatin	336 (24.5)
Gemcitabine + Carboplatin	86 (6.2)
Cisplatin + Paclitaxel	54 (3.9)
Adriamycin + Cyclophosphamide	1 (0.07)
Capeox + Capecitabine	145 (10.6)
Gemcitabine + Cisplatin	42 (3.0)
Triple regimen	
Velcade+ Revlimid+ Dexamethasone	6 (0.4)
Bortezomib+ Thalidomide+ Dexamethasone	12 (0.8)
Bortezomib+ Cyclophosphamide+ Dexamethasone	8 (0.5)
Quadruple regimen	
Cyclophosphamide+ Doxorubicin+ Vincristine+ Prednisolone	7 (0.5)
Doxorubicin+ Bleomycin+ Vinblastine+ Dacarbazine	9 (0.6)
Other Treatment Modalities*	207 (15.1)
Supportive treatment	
Anti-emetic	628 (45.9)
Antacid	580 (42.4)
Anti-inflammatory	625 (45.7)
Anti-histamine	496 (36.3)

Other Treatment Modalities*- Sorafenib/Lenvatinib, Only Surgery, Surgery+ Radiation, Surgery+ Chemotherapy+ Radiation, PTBD Or Stenting, Only Radiation, Hormone Replacement Therapy.

Among patients receiving chemotherapy, the majority completed two cycles (30.67%), followed by three cycles (28.69%). Regarding single-drug regimens, Paclitaxel and Carboplatin were the most frequently prescribed agents. For two-drug combinations, Paclitaxel with Carboplatin was the most common pairing, followed by CapeOX (Capecitabine and Oxaliplatin). More complex regimens involving three or four drugs were less frequent, including combinations such as Bortezomib, Thalidomide, and Dexamethasone, or Doxorubicin, Bleomycin, Vinblastine, and Dacarbazine. In addition to chemotherapy, approximately 15% of patients received other forms of therapy such as targeted therapy, surgery, or multimodal combinations.

### Supportive therapies

Anti-emetics (45.97%), anti-inflammatory drugs (45.75%), antacids (42.45%), and antihistamines (36.31%) were the most commonly administered supportive therapies (**Table 4**). These therapies were integral to improving patient quality of life during treatment.

### Mortality and outcomes

In-hospital mortality was similar between rural (4.82%) and urban (4.22%) groups (p = 0.661). Readmission rates were also not significantly different, with 75.1% of patients readmitted fewer than five times (p = 0.148) (**Table 1**).

### Trend analysis of year-wise cancer distribution (2021–2023)

**Supplementary Table S2** depict a clear upward trend in cancer incidence, rising from 140 cases in 2021 to 710 in 2023. Head and neck cancers consistently remained the most prevalent, while lung and breast cancers showed a sharp increase over the three years. Colorectal cancers also maintained a high frequency, whereas gallbladder and gynecological cancers showed a gradual decline. Notably, emerging cases of pancreatic, prostate, myeloma, and soft-tissue sarcomas in 2023 may reflect advancements in diagnostic capabilities. Overall, the data indicate a growing burden of thoracic and gastrointestinal malignancies in recent years.

## Discussion

To our knowledge, this is the first study to provide a snapshot regarding the urban-rural disparity and determine the healthcare utilization among cancer patients in Rajasthan. This study presents a detailed comparative analysis of the clinicodemographic characteristics of rural and urban patients, with a focus on identifying patterns of healthcare utilization, disease presentation, and treatment outcomes.

The predominance of rural patients in the study population reflects the geographic distribution and catchment area of the healthcare facility under investigation. Interestingly, these findings contrast with global patterns and earlier studies reporting a higher cancer burden in urban areas (15, 16). More recent evidence from the United States, however, has demonstrated higher overall cancer incidence among rural populations compared with urban counterparts, particularly for tobacco and infection associated malignancies, highlighting that rural and urban disparities in cancer burden persist even in developed settings (17). In India, similar trends have been observed, with rural populations, particularly in eastern states such as West Bengal and Bihar and northern states such as Uttar Pradesh and Rajasthan, showing higher rates of late-stage cancer presentation (18). Recent hospital and population-based cancer registry reports from Bihar and Uttar Pradesh further support this pattern, documenting a rising cancer burden in rural areas and a greater prevalence of advanced stage and site-specific cancers, particularly cervical malignancies among rural women (19, 20).

Consistent with these findings, several studies from India, China, and the United States highlight persistent disparities in rural cancer care, characterized by limited healthcare infrastructure, delayed diagnosis, and restricted access to specialized oncology services (21–23). These discrepancies likely stem from healthcare inaccessibility, socioeconomic constraints, and lower educational attainment, all of which contribute to diagnostic delays and a disproportionate cancer burden in rural communities.

According to the demographic statistics, the majority of the study population hailed from rural areas, with 54.15% of the cancer patients being male and 45.84% being female. This gender distribution is consistent with global cancer data, which show that certain cancer forms are more common among men (24). The average age of the patients was 53.78 years, indicating that cancer cases were predominant among the older population, which is consistent with the general pattern that shows an increase in cancer incidence with age (25, 26).

Tobacco use was significantly more prevalent among rural patients (16.8%) compared to their urban counterparts (10.4%) (p = 0.006). This pattern aligns with nationwide data from the Global Adult Tobacco Survey (GATS-2, 2017), which reported higher smokeless tobacco use in rural India, and is consistent with prior research, including Gayatri, et al. (2023), which highlighted the entrenched cultural acceptability of tobacco in rural settings, especially among males, compounded by poor health literacy and limited access to cessation services (27, 28). The public health implications are far-reaching, necessitating culturally sensitive, locally adapted tobacco control interventions that integrate behavioral change, policy enforcement, and health education.

One of the most clinically significant findings of this study was the advanced stage of disease at presentation among rural patients. A considerably higher proportion of rural patients (56.1%) presented with Stage 4 disease compared to 47.7% of urban patients (p = 0.047). This pattern aligns with a population-based registry study from Varanasi district (Uttar Pradesh, North India) (15), which reported that rural cancer patients were more likely to be diagnosed at distant stage than their urban counterparts. It is also consistent with a systematic review by Afshar et al., which found that late-stage diagnosis is more common among rural patients due to delays in seeking medical care and limited access to diagnostic facilities (29). The importance of early detection in improving cancer prognosis is underscored by the high prevalence of late-stage diagnosis in rural areas, which highlights the urgent need for improved screening and awareness programs in these regions. In the adjusted analysis, rural residence remained significantly associated with late-stage presentation, indicating that this disparity persisted even after controlling for age, sex, tobacco use, and treatment modality.

Economic burden significantly exacerbates rural-urban disparities in cancer care. Nationally, cancer treatment pushes 55–65% of households into poverty, with rural families incurring 1.8-times higher catastrophic costs due to drugs, travel, and lost income (30). In Rajasthan, the Mukhya Mantri Nishulk Dava Yojana (Free Cancer Medicine Scheme) addresses this burden by supplying 39 essential chemotherapy drugs free of cost through public hospitals, substantially lowering patients’ out-of-pocket expenses. Despite this, urban patients in our study received more combination regimens, likely due to uncovered radiotherapy and travel expenses. Integrating free diagnostics, tele-oncology, and Ayushman Bharat–Chiranjeevi Yojana coverage is essential to eliminate economic barriers and improve equitable outcomes.

Readmission rates also did not differ significantly between the two groups, though rural population had slightly higher readmission rate (>5 times). A finding by Echere et al. (2024), demonstrated a higher frequency of readmissions in rural patients due to limited healthcare access, higher prevalence of chronic conditions, and socioeconomic challenges (31). In the current study setting, the presence of a structured follow-up mechanism, potentially supported by digital health initiatives and community health workers have mitigated this disparity. This highlights the importance of robust discharge planning and patient follow-up to ensure treatment adherence and reduce preventable readmissions, especially in resource-limited rural settings.

The district-wise analysis revealed that Alwar had the highest number of cases, followed by Jaipur, Bharatpur, and Sikar. The distribution of cancers and the number of cases in these districts represent the regional cancer burden. A study by Freddie et al. highlights the significance of district-level cancer registries for successful healthcare planning (32).

There was a notable difference in the distribution of treatment regimens among patients living in rural and urban areas. A wide range of chemotherapy combinations, with carboplatin and paclitaxel being the most common double regimen treatment. The use of single, double, triple, and quadruple chemotherapy regimens is consistent with current cancer treatment guidelines, which frequently include combination medicines to increase efficacy (33). In our study, patients living in urban areas tended to receive a combination of therapies, such as chemotherapy and radiation, while those in rural areas predominantly received single-regimen treatments. This disparity can be attributed to differences in healthcare infrastructure and availability of specialized oncology services. A study by Bhatia S et al. highlighted the challenges faced by patients in rural areas when it comes to accessing advanced cancer treatments. As a result, these patients often experience less favorable outcomes (23). Supportive therapy was frequently used, with anti-emetic, anti-inflammatory, and antacid drugs being the most popular. The widespread use of supportive therapy emphasizes the necessity of controlling cancer treatment side effects to improve patient quality of life (34).

The in-hospital mortality rate was higher among patients from rural areas (4.82%) compared to those from urban areas (4.22%). This is consistent with the results of a study conducted by Blake KD et al. (2017) and Afshar N et al. (2019) revealed that rural cancer patients experienced higher mortality rates (29, 35). The study by Tedder T et al. (2017) further reinforces these findings, emphasizing that patients in rural areas frequently exhibit more advanced disease and face challenges in accessing palliative care services (36).

### Limitations

This study is not without limitations. First, it was a single-center retrospective analysis, which may limit the generalizability of findings to the broader population of Rajasthan. Second, missing or incomplete documentation in retrospective data could have introduced minor classification bias. Third, the predominance of rural participants and the hospital’s tertiary-care referral pattern may have introduced selection and referral bias, leading to overrepresentation of certain geographic regions. Despite inclusion of a large cohort from multiple districts, these factors could have influenced the observed urban–rural distribution. Future multicentric, population-based registries with prospective follow-up are warranted to validate and expand these observations.

## Conclusion

This study provides a comprehensive epidemiological assessment of cancer patients from a tertiary care center in Rajasthan, revealing marked urban–rural disparities in disease stage, healthcare access, and treatment patterns. The predominance of rural patients, late-stage presentation, and higher tobacco use reflect critical gaps in early detection and preventive care. Targeted interventions such as deployment of mobile cancer screening units, district-level oncology clinics, and community-based tobacco cessation and awareness programs are urgently needed. Strengthening regional cancer registries and tele-oncology networks can further enhance early diagnosis and continuity of care in underserved areas. These findings underscore the need for evidence-driven policy measures to promote equitable access and improved cancer outcomes across diverse populations.

## Data Availability

The raw data supporting the conclusions of this article will be made available by the authors upon genuine request to the corresponding authors, without undue reservation.
