# Correction: Epidemiological profile of patients with malignant neoplasm admitted to a tertiary care center in India: a retrospective cross-sectional study

**DOI:** 10.3389/fonc.2026.1783850

**Published:** 2026-01-28

**Authors:** Sourav Debnath, Pusparghya Pal, Anurag Kumar Singh, Shivang Mishra, Sumit Rajotiya, Manashi Ghosh, Prashant Nakash, Sachin Kumar, Roshni Singh, Govind Sharma, Mahaveer Singh, Deepak Nathiya, Balvir Singh Tomar

**Affiliations:** 1 Department of Pharmacy Practice, Nims Institute of Pharmacy, Nims University Rajasthan, Jaipur, India; 2 Department of Medical Oncology, National Institute of Medical Sciences and Research, Nims University Rajasthan, Jaipur, India; 3 Department of Management, Nims Institute of Management and Commerce, Nims University Rajasthan, Jaipur, India; 4 Department of Endocrinology, National Institute of Medical Sciences and Research, Nims University Rajasthan, Jaipur, India; 5 Institute of Pediatric Gastroenterology and Hepatology, National Institute of Medical Sciences and Research, Nims University Rajasthan, Jaipur, India

**Keywords:** cancer epidemiology, rural-urban disparities, treatment access, tobacco use, oncology outcomes, Rajasthan

In the published article, there was an error in the author affiliations.

The affiliations were erroneously published as follows:

^1^Department of Pharmacy Practice, National Institute of Medical Sciences and Research, Jaipur, Rajasthan, India

^2^Department of Oncology, National Institute of Medical Sciences and Research, Jaipur, Rajasthan, India

^3^Clinical Research Division, Patanjali Research Foundation, Trust, Haridwar, Uttarakhand, India

^4^Department of Management, NIMS Institute of Management and Commerce, NIMS University, Jaipur, Rajasthan, India

^5^Department of Endocrinology, National Institute of Medical Sciences and Research, Jaipur, Rajasthan, India

^6^Institute of Pediatric Gastroenterology and Hepatology, National Institute of Medical Sciences and Research, Jaipur, Rajasthan, India

The correct affiliations are as follows:

Sourav Debnath^1^, Pusparghya Pal^1*^, Anurag Kumar Singh^1*^, Shivang Mishra^1^, Sumit Rajotiya^1^, Manashi Ghosh^2^, Prashant Nakash^1^, Sachin Kumar^1^, Roshni Singh^1^, Govind Sharma^3*^, Mahaveer Singh^4^, Deepak Nathiya^1^, Balvir Singh Tomar^5^

^1^Department of Pharmacy Practice, Nims Institute of Pharmacy, Nims University Rajasthan, Jaipur, India

^2^Department of Medical Oncology, National Institute of Medical Sciences and Research, Nims University Rajasthan, Jaipur, India

^3^Department of Management, Nims Institute of Management and Commerce, Nims University Rajasthan, Jaipur, India

^4^Department of Endocrinology, National Institute of Medical Sciences and Research, Nims University Rajasthan, Jaipur, India

^5^Institute of Pediatric Gastroenterology and Hepatology, National Institute of Medical Sciences and Research, Nims University Rajasthan, Jaipur, India

The original version of this article has been updated.

