# Supplementary material for: Epidemiological profile of patients with malignant neoplasm admitted to a tertiary care center in India: a retrospective cross-sectional study
Source: Front Oncol. 2025 Dec 8;15:1636807. doi: 10.3389/fonc.2025.1636807 (PMC12722929; doi:10.3389/fonc.2025.1636807)
Supplement: Supplementary file 1 [file Table1.docx]

**Supplementary Table S1:** Multivariable logistic regression analysis for factors associated with late-stage cancer presentation

| **Variable** | **B** | **Adjusted OR** | **95% CI for Exp(B)** | **p-value** |
| --- | --- | --- | --- | --- |
| **Residence (**Rural vs. Urban) | 0.315 | 1.37 | 1.06 – 1.78 | **0.018** |
| **Age** (years) | -0.002 | 0.998 | 0.99 – 1.01 | 0.665 |
| **Gender** (Female vs. Male) | -0.289 | 0.75 | 0.60 – 0.94 | **0.012** |
| **Tobacco use** (Yes vs. No) | 0.150 | 1.16 | 0.84 – 1.61 | 0.367 |
| **Treatment modality (ref category: Surgery)**  Chemotherapy  Radiotherapy  Others* | 0.208  0.272  0.693 | 1.23  1.31  2.00 | 0.92 – 1.64  0.96 – 1.79  1.36 – 2.94 | 0.152  0.092  0.073 |
| **OR:** Odds ratio.  *Others include hormone therapy, conservative management, and palliative care. Advanced stage was defined as Stage IV. Adjusted for residence, age, gender, tobacco use, and treatment modality. Statistically significant at p < 0.05. | | | | |

**Supplementary Table S2:** Year-wise distribution of different malignancies (2021-2023)

| **Cancer site** | **Year 2021**  **[n (%)] 140** | **Year 2022**  **[n (%)] 516** | **Year 2023**  **[n (%)] 710** |
| --- | --- | --- | --- |
| 1. Ca Breast | 6 (4.3) | 44 (8.5) | 62 (8.7) |
| 2. Ca Lung | 9 (6.4) | 109 (21.1) | 122 (17.2) |
| 3. Ca Head & Neck | 31 (22.1) | 117 (22.7) | 135 (19.0) |
| 4. Ca Colorectal | 14 (10.0) | 49 (9.5) | 66 (9.3) |
| 5. Leukaemia | 5 (3.6) | 6 (1.2) | 9 (1.3) |
| 6. Ca Liver | 9 (6.4) | 11 (2.1) | 10 (1.4) |
| 7. Ca Pancreas | 0 | 5 (1.0) | 13 (1.8) |
| 8. Ca Bone Sarcoma | 2 (1.4) | 2 (0.4) | 11 (1.5) |
| 9. Ca Gallbladder & biliary Tract | 15 (10.7) | 34 (6.6) | 35 (4.9) |
| 10. Ca Cervix | 6 (4.2) | 10 (1.9) | 11 (1.5) |
| 11. Ca Endometrium | 2 (1.4) | 4 (0.8) | 10 (1.4) |
| 12. Ca Ovary | 17 (12.1) | 30 (5.8) | 48 (6.8) |
| 13. Ca Urinary Bladder | 3 (2.1) | 15 (2.9) | 20 (2.8) |
| 14. Ca Kidney | 0 | 8 (1.6) | 13 (1.8) |
| 15. Melanoma | 3 (2.1) | 6 (1.2) | 7 (0.9) |
| 16. Ca Soft-tissue sarcoma | 3 (2.1) | 6 (1.2) | 17 (2.4) |
| 17. Ca Stomach | 4 (2.9) | 19 (3.7) | 22 (3.1) |
| 18. Ca Prostate | 1 (0.7) | 16 (3.1) | 26 (3.7) |
| 19. Ca Esophagus | 2 (1.4) | 15 (2.9) | 28 (3.9) |
| 20. Ca Testis | 2 (1.4) | 2 (0.4) | 6 (0.8) |
| 21. Ca Duodenum | 1 (0.7) | 2 (0.4) | 9 (1.3) |
| 22. Ca Thyroid | 4 (2.9) | 3 (0.6) | 0 |
| 23. Ca Myeloma | 1 (0.7) | 3 (0.6) | 22 (3.1) |
| 24. Other Malignances* | 0 | 0 | 8 (1.1) |
| ***Other Malignances*-*** *Brain Tumor, Ca Penis, Ca Adrenal Gland, Carcinoma of unknown primary.* | | | |
